# Light Therapy for Older People with Depressive Symptoms: Systematic Review and Meta-Analysis

**DOI:** 10.3390/jcm13226982

**Published:** 2024-11-20

**Authors:** Ji-Woo Seok, Jung-Dae Kim

**Affiliations:** Digital Health Research Division, Korea Institute of Oriental Medicine, 1672 Yuseong-daero, Yuseong-gu, Daejeon 34054, Republic of Korea; suk6124@kiom.re.kr

**Keywords:** light therapy, depressive symptom, meta-analysis, light intensity, light spectrum, older people

## Abstract

**Background/Objectives:** Light therapy has emerged as a promising non-pharmacological treatment for depressive symptoms. This meta-analysis aims to evaluate the effectiveness of light therapy specifically for depressive symptoms in elderly populations, with a focus on how different light intensities and spectra influence treatment outcomes. **Methods:** A systematic search targeting studies on light therapy for depressive symptoms in older adults was performed across multiple databases, including PubMed, Google Scholar, PsycINFO, and EMBASE, covering studies from database inception until July 2024. A total of 565 records were identified, with 461 studies remaining after removing duplicates. Following the screening of titles and abstracts, 54 studies underwent full-text review, resulting in the inclusion of 22 studies with a total of 1290 participants (687 in the intervention group and 603 in the control group). **Results:** The overall effect size for light therapy on depressive symptoms was moderate (Hedges’ g = 0.525, *p* < 0.001). Higher light intensities (10,000 lux and above) demonstrated significantly greater effectiveness compared to lower intensities. White light had the most substantial effect, while bluish light showed moderate efficacy. Significant heterogeneity was observed across studies (I^2^ = 80.459%), indicating variability in treatment outcomes based on study design, intensity, and light spectrum. **Conclusions:** This meta-analysis confirms that light therapy is an effective treatment for reducing depressive symptoms in older adults, particularly at higher intensities and with specific light spectra such as white light. Given the heterogeneity in results, future research should focus on optimizing treatment parameters to enhance clinical outcomes within this population.

## 1. Introduction

Depression among older adults is a significant public health issue that often goes unrecognized and untreated, leading to considerable distress. This condition places a heavy strain on both families and caregiving institutions, as it incapacitates individuals who might otherwise be healthy and able to function independently [1]. According to the DSM-5, major depressive disorder (MDD) is defined by the presence of at least five depressive symptoms, such as persistent sadness, fatigue, or changes in sleep and appetite, lasting for at least two weeks. In contrast, subthreshold depressive symptoms, though not meeting the full MDD criteria, still cause significant emotional distress and impair daily functioning. Both conditions are often collectively termed “depression” due to their similar impacts on emotional and functional well-being [2,3]. Depression in the elderly is prevalent and may persist from earlier in life or emerge after age 60. A review study included 20 studies with a total of 18,953 participants and found a global prevalence of major depression in the elderly of 13.3% (95% CI: 8.4–20.3%). In a subgroup analysis, prevalence was 11.9% in women (95% CI: 7.6–18.6%) and 9.7% in men (95% CI: 5.2–17.3%), with no significant difference between sexes [4]. A recent meta-analysis estimated the prevalence of depressive symptoms among older adults to be 31.74% (95% CI: 27.90, 35.59), with rates ranging from 17.05% in developed countries to 40.78% in developing countries [5].

Depression in older adults is not merely an emotional issue but has serious implications for overall quality of life, a phenomenon consistently observed across various cultural contexts [6,7,8]. Specifically, depression adversely affects functional status in essential daily activities required for independent living, including both basic and instrumental daily functions. These activities encompass critical self-care tasks, such as financial management, medication adherence, and meal preparation, as well as higher-order social engagement [6,8]. Recent studies highlight the specific challenges in financial capacity among older adults with depression. Financial capacity, which includes essential skills like managing bills, understanding currency, and making financial decisions, is often severely compromised in individuals with depressive symptoms. This impairment can significantly hinder independent living and increase vulnerability to financial exploitation, further stressing the importance of targeted assessments in this area [9]. Studies indicate that even in older adults without neurocognitive disorders or with only mild depressive symptoms, depressive symptom are strongly associated with diminished daily functioning, with greater depressive severity leading to increased limitations [10,11]. This vicious cycle not only weakens individual independence but also heightens caregiver burden and raises social costs within healthcare systems [12]. Therefore, maintaining functional independence and improving quality of life in older adults necessitates effective interventions aimed at managing and reducing depression.

Psychotherapy and antidepressants are commonly used to alleviate depressive symptoms in the elderly [13,14]. However, older patients are at a higher risk of experiencing side effects from antidepressants compared to other age groups, and psychotherapy is limited by the shortage of psychologists and psychiatrists [15,16]. For these reasons, non-pharmacological treatments that are more accessible and have fewer side effects have been developed as complementary approaches for treating depression in elderly patients. Light therapy, often referred to as phototherapy, involves controlled exposure to sunlight or specific types of artificial light to address various medical conditions by using targeted wavelengths [17]. This light is often delivered via a light box, which includes fluorescent tubes, a reflector, and a diffusing screen, or through fluorescent ceiling units [18,19]. 

People showing depressive symptoms frequently exhibit atypical symptoms linked to circadian rhythm disruptions, such as irregular sleep-wake cycles, altered social rhythms, mood fluctuations throughout the day, and changes in hormonal and core body temperature patterns [20,21]. Light therapy has been explored as a treatment for non-seasonal depression due to its potential to stabilize these disrupted rhythms and improve mood regulation [22]. By stimulating the suprachiasmatic nucleus (SCN), the brain’s circadian clock, light therapy is thought to normalize the sleep-wake cycle and regulate melatonin secretion, which may be particularly beneficial for individuals experiencing such disruptions [20,22,23]. This mechanism is also believed to positively influence mood, sleep patterns, and hypothalamic-pituitary-adrenal axis activity, similar to its effects on patients with affective disorder [20,24,25]. However, the exact mechanisms by which light therapy exerts its effects remain unclear.

Light therapy offers several advantages in treating depression, particularly for older adults. First, it is a non-invasive and safe treatment option that avoids the potential side effects associated with antidepressants, which can be a significant concern for the elderly. Studies have shown that light therapy can effectively improve mood and reduce depressive symptoms in both seasonal affective disorder (SAD) and non-seasonal depression, making it versatile across different forms of depression [22,23,26]. Another benefit is that light therapy helps to regulate circadian rhythms, which are often disrupted in older individuals, contributing to both sleep disturbances and mood disorders. By resynchronizing these rhythms, light therapy can lead to improvements in both sleep quality and depressive symptoms [21,26]. Furthermore, light therapy has shown a rapid onset of action compared to antidepressants, which can take weeks to become effective. Some studies suggest that improvements can be seen as early as within a few days of starting light therapy [27,28]. Lastly, light therapy is cost-effective and easily accessible, making it an attractive alternative for the elderly who may have difficulty accessing frequent psychotherapy sessions or managing medication regimens [19,27].

Building on these findings, recent meta-analytic research has further evaluated the efficacy of light therapy in improving depressive symptoms among older adults. A meta-analysis by Chang (2018) demonstrated that light therapy significantly reduced depression severity when compared to placebo or dim light, with an effect size of 0.442 (95% CI: 0.174–0.709, *p* = 0.001) [29]. Another meta-analysis by Aini (2024) found that light therapy has a small to moderate effect in reducing depression among people living with dementia, with an overall effect size for depression reduction of Hedges’ g = −0.46, indicating a modest but significant improvement in depressive symptoms [30]. These findings suggest that light therapy can be an effective non-pharmacological intervention for treating non-seasonal depression in elderly adults, offering a promising alternative to traditional treatments such as psychotherapy and antidepressants.

Despite these promising results, previous meta-analyses on light therapy for depression have limitations due to the small number of studies included, with only 8 to 9 studies analyzed in each [29,30]. This limited sample size restricts the ability to draw robust conclusions, indicating the need for further studies to increase the reliability of these findings. Furthermore, existing meta-analyses have highlighted several issues, including observed heterogeneity among studies, which suggests variability in outcomes across trials, and signs of publication bias. These limitations imply that additional research is necessary to confirm the robustness of light therapy’s effects on depression. Moreover, to maximize the potential benefits of light therapy, it is essential to identify factors contributing to this heterogeneity, such as light intensity, duration of exposure, and optimal timing of administration. Previous meta-analyses have been unable to conduct comprehensive meta-regression analyses to examine these potential moderators due to the limited number of studies included (fewer than 10), thereby limiting the ability to identify specific parameters that may enhance therapeutic outcomes [29,30].

To address these limitations, the present study aims to include a larger pool of studies to evaluate the overall effects of light therapy on depressive symptoms and to conduct meta-regression analyses to identify moderators influencing the efficacy of light therapy. By refining specific treatment parameters, this study seeks to provide more reliable and individualized recommendations for implementing light therapy as a non-pharmacological treatment for depression in older adults.

## 2. Methods

### 2.1. Study Design 

We conducted the meta-analysis and meta-regression analysis and presented the findings following the Preferred Reporting Items for Systematic Reviews and Meta-Analyses (PRISMA) guidelines [31]. Additionally, this review was registered in the International Prospective Register of Systematic Reviews (PROSPERO) under the registration number CRD42023401981 [32].

### 2.2. Inclusion and Exclusion Criteria

The predetermined inclusion criteria was based on the following: (1) Population: elderly individuals diagnosed with depression or considered at risk for depression based on validated depression assessment tools; (2) Intervention: light therapy; (3) Comparison: control groups that did not receive any intervention (e.g., no treatment, waitlist control), or those receiving only a placebo or usual care; (4) Outcomes: changes in depressive symptoms assessed using objective or subjective measures; and (5) Study Design: randomized controlled trials and controlled clinical trials. The exclusion criteria included: (1) studies with irrelevant topics, populations, or study designs, (2) duplicate publications, (3) studies that did not report depression outcomes, and (4) literature reviews, case reports, and qualitative studies.

### 2.3. Data Search and Selection Process

Two independent authors conducted a literature search across electronic databases, including PubMed, MEDLINE, the Cochrane Central Register of Controlled Trials (CENTRAL), EMBASE, Web of Science, PsycINFO, Google Scholar and ProQuest Dissertations. A comprehensive literature search was performed across multiple dates to ensure the inclusion of the most recent studies. The initial search was conducted between December 2023 and January 2024, and a follow-up search was carried out between June and July 2024 to capture any new publications that may have emerged in the interim. 

We conducted a literature search using targeted combinations of keywords to enhance relevance, including: (Bright light therapy OR BLT OR Phototherapy OR Light therapy) AND (depression OR depressive symptom OR mood) AND (old adults OR elders OR geriatric) AND (Placebo OR sham intervention OR Waitlist OR TAU OR treat as usual OR no intervention OR CAU OR care as usual) AND (RCT OR randomized control trial OR crossover design OR pretest and posttest). In addition, we manually checked the reference lists of the identified studies and relevant articles suggested by meta-analyses and systematic reviews. No restrictions were placed on the country of publication, or the participants’ gender or race. After removing duplicates from the literature gathered through electronic databases and manual searches, the titles and abstracts were screened to identify potentially relevant studies. We then assessed the full texts of these studies according to the inclusion and exclusion criteria to determine the final selection. Two researchers independently carried out selection process. In cases of disagreement, they re-assessed the studies based on the inclusion and exclusion criteria and resolved any differences through team discussion.

### 2.4. Data Extraction and Analysis

We developed a coding framework based on relevant prior literature, and two research assistants extracted the necessary information according to this framework. The following study characteristics were extracted: title, author, publication year, participant characteristics (i.e., diagnosis and age), sample size, intervention characteristics (light intensity, device, type of light, frequency, duration, and length of therapy), control type, measurement tool, and overall depression scores before and after the intervention. In cases where post-intervention scores were collected at multiple follow-up intervals, we prioritized the assessment conducted immediately following the intervention. The extracted data were independently reviewed, and any discrepancies were resolved through consensus.

### 2.5. Quality Assessment of Included Studies

Two independent researchers performed a thorough full-text review and appraised the quality of the included studies using the revised Cochrane risk of bias tool for randomized controlled trials (RoB 2.0) [33]. The quality assessment covered several key domains, including: bias arising from the randomization process, bias due to deviations from intended interventions, bias due to missing outcome data, bias in the measurement of the outcome, and bias in the selection of the reported result. Each domain was classified as having a high, some concerns or low risk of bias. The quality assessment findings were independently verified, and any differences between the reviewers were addressed through discussion. When disagreements persisted, a third researcher was consulted to facilitate consensus.

### 2.6. Statistical Analysis

We conducted a random effects meta-analysis to synthesize the results across studies using JASP version 0.19.0.0 (Jeffreys’s Amazing Statistics Program). The model was chosen to account for potential heterogeneity between studies, which may arise from differences in study design, populations, or intervention methods [34]. We used Hedges’ g to measure effect size, as it adjusts for the bias in Cohen’s d, which tends to overestimate effect size in small samples [35]. Many studies in this meta-analysis had small sample sizes, so Hedges’ g was applied for accuracy [36,37]. Effect sizes were categorized as small (<0.15), medium (0.40–0.74), and large (>0.75) [38]. We interpreted effect sizes with 95% confidence intervals, considering effects significant if the interval excluded 0 [36].

The heterogeneity of the included studies was assessed using the Q statistic and the I^2^ index. A significant Q statistic (*p* < 0.001) indicated the presence of heterogeneity, and the I^2^ index was used to quantify the proportion of variance attributable to between-study differences, with values over 75% considered substantial heterogeneity [39,40]. To investigate the origins of heterogeneity, a meta-regression analysis was performed, assessing moderating effects of study-level characteristics like participant and intervention/control characteristics [41]. The restricted maximum likelihood (REML) method was used to estimate model parameters [42]. An omnibus test of regression coefficients and the Q-statistic were used to confirm significant effects across studies [43].

We evaluated publication bias by examining the funnel plot’s symmetry, where individual studies’ estimated effect sizes were plotted on the x-axis [44]. To further detect any potential publication bias, we applied Kendall’s τ rank correlation and Egger’s regression tests [45]. Additionally, a Fail-safe N test was used to determine how many unpublished studies would be needed to negate the observed effect size, assessing the robustness of the results [46].

## 3. Results

### 3.1. Selection of Studies

**Figure 1:**
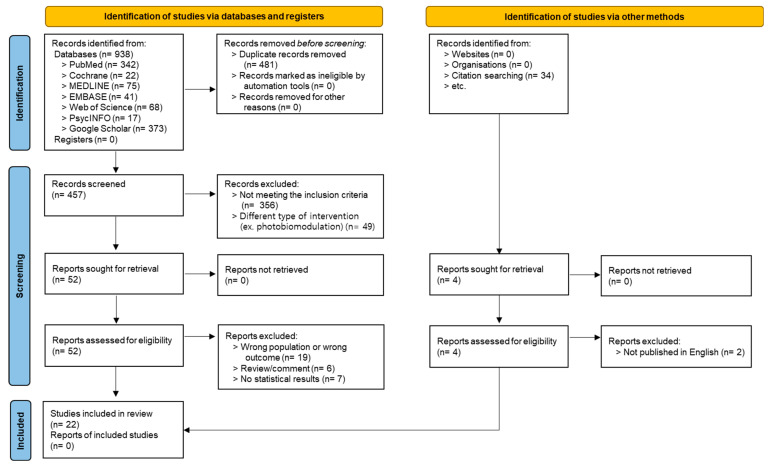
Flowchart of study selection.

A comprehensive search resulted in the identification of 938 studies from database searches, with an additional 34 records identified via other methods, including websites and citation searching. After removing 481 duplicates, 457 records remained for title and abstract screening. Of these, 405 studies were excluded for not meeting the inclusion criteria (356 studies) or due to a different type of intervention, such as photobiomodulation or light modulation interventions (49 studies). The full texts of 52 articles were reviewed for eligibility. However, 34 studies were excluded for various reasons: (1) 19 studies were excluded because they involved the wrong population (i.e., not older patients) or assessed the wrong outcome (i.e., lacking a depression measure), (2) four review articles and two trial protocols were excluded, (3) seven studies were excluded because they either consisted of a single arm or provided only post-intervention statistics for the experimental and control groups, making it impossible to extract pre- and post-intervention means and standard deviations for each group, and (4) two studies were excluded because they were not published in English. Ultimately, 22 studies were included in the final meta-analysis (Figure 1).

### 3.2. Characteristics of the Studies

A total of 22 studies were included in the meta-analysis, and their characteristics are summarized in Table 1. These aspects include the authors, year of publication, participant characteristics (i.e., diagnosis and age), the number of participants in each study, the type of control group used, and the characteristics of the light therapy interventions. Specifically, the intervention details cover the light intensity (measured in lux), the device and type of light used (i.e., spectrum), the frequency of the intervention (number of sessions per day), the duration of each session (measured in minutes), and the total period of the intervention (in days).

These studies were published between 2001 and 2022, focusing primarily on older adults with various diagnoses such as dementia, Alzheimer’s disease, major depressive disorder, Parkinson’s disease, and depression in aging populations. The final quantitative analysis included 1290 participants from 22 trials (intervention: 687, control: 603). The sample sizes across the studies ranged from 10 to 96 participants, with ages varying from mid-60 s to late 80 s.

The interventions in the included studies utilized light therapy with varying intensities, devices, and types of light. Light intensities ranged from 30 to 14,000 lux, using various devices such as light boxes, ceiling-mounted LED panels, and head-mounted devices. The types of light used included bright white, bluish-white, blue-enriched, and full-spectrum light. Interventions were typically conducted once per day, with session durations ranging from 30 to 540 min per day (average: 108.75 ± 137.98 min).The average treatment duration across studies was 33.55 ± 24.49 days (range: 5–90 days). Control groups in these studies generally received either placebo light treatments, dim light, or no treatment. Various measurement tools were used to assess depression outcomes, including the Cornell Scale for Depression in Dementia, Hamilton Depression Rating Scale, Beck Depression Inventory, and the Geriatric Depression Scale.

### 3.3. Quality Assessment Results

We conducted a risk of bias analysis for the 22 studies using the RoB 2.0. Table S1 presents a summary of the risk of bias for each study. Table S1 presents a summary of the risk of bias for each study. According to our assessment, none of the studies were evaluated as having a low overall risk of bias, with all studies classified as having some concerns or high risk in one or more areas. The primary areas of concern were found in the randomization process and deviations from intended interventions. Nearly all studies lacked sufficient clarity regarding the randomization process, particularly in the specifics of allocation concealment. This was a common issue, with many studies not providing adequate information about whether participants or personnel were blinded appropriately, leading to some concerns in the majority of studies. Additionally, the handling of missing outcome data raised concerns in many studies. Several studies reported participant dropout rates, with figures ranging from small percentages to more significant levels. However, few studies adequately addressed how missing data were handled, whether through intention-to-treat analysis or other methods, which led to some concerns about the influence of incomplete data on the results.

In contrast, the measurement of outcomes and selection of reported results were generally well-executed. Most studies ensured proper blinding of outcome assessors, although a few did not clearly describe their blinding procedures. The risk of selective reporting was relatively low, as most studies appeared to report all predefined outcomes comprehensively, mitigating concerns in these areas. Overall, the primary issues identified across the 22 studies were concentrated in the early stages of trial design, particularly in the randomization and intervention processes, while outcome measurement and reporting were generally well-managed.

### 3.4. Effect Size of Light Therapy on Depressive Symptom 

**Figure 2:**
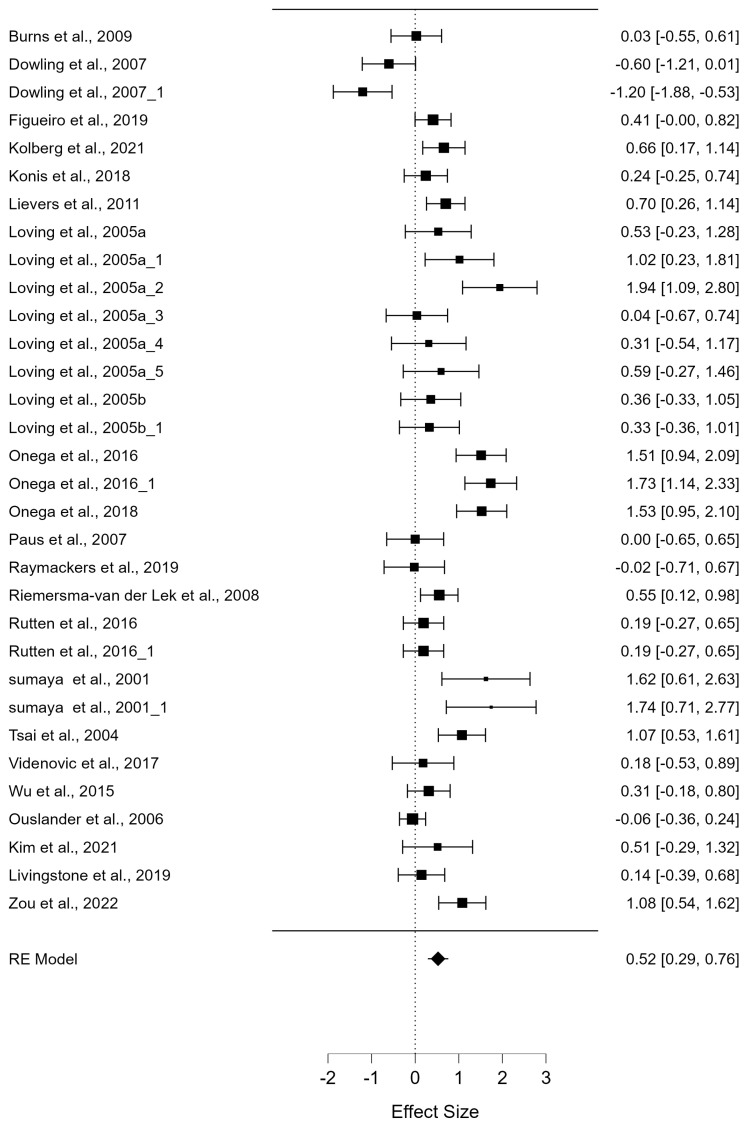
The effect of light therapy on depressive symptom [24,47,48,49,50,51,52,53,54,55,56,57,58,59,60,61,62,63,64,65,66,67].

The meta-analysis examining the effect of light therapy on depressive symptoms revealed significant findings. The overall effect size for the intervention was estimated at 0.525, which was statistically significant (z = 4.414, *p* < 0.001, with a 95% confidence interval (CI) of [0.292, 0.758]). This indicates a medium effect size according to conventional interpretations of effect size (Hedges’ g). The result suggests that light therapy has a meaningful impact on reducing depressive symptoms (Figure 2).

The heterogeneity across studies was also significant, as demonstrated by the residual heterogeneity test Q(31) = 140.313, *p* < 0.001. This high heterogeneity indicates substantial variability in effect sizes between the studies, implying that different factors (e.g., intensity of light, duration, or participant characteristics) might be contributing to these differences in outcomes. Further, the estimates for residual heterogeneity were τ^2^ = 0.347 and τ = 0.589, with an I^2^ statistic of 80.459%, showing that about 80% of the total variation in effect sizes is due to between-study differences rather than sampling error. The H^2^ statistic was 5.117, confirming a high level of heterogeneity.

### 3.5. Moderator Effects of Light Therapy and Subgroup Analyses 

In this meta-analysis, significant heterogeneity was found across studies, leading us to perform subgroup analyses and meta-regression to explore the factors contributing to this variability. We included participant type, intervention type characteristics (e.g., device, light intensity, and light spectrum type), protocol characteristics (e.g., total duration for light therapy and length of therapy per session), and control type as moderators in the meta-regression to assess their influence on the effect of light therapy in Table 2. These analyses helped identify how these factors contributed to the observed heterogeneity and their role in explaining the varying effects of light therapy across studies.

Participant type. Based on the results of the meta-regression, the analysis of participant type showed no significant differences in the overall effect size of light therapy between the reference group, which was the dementia group, and the depression group, Parkinson groups. Specifically, the depression group had an estimated effect size of 0.253 (z = 0.965, *p* = 0.334), indicating no statistically significant difference compared to the dementia group. Similarly, the Parkinson’s group had an estimated effect size of −0.404 (z = −1.181, *p* = 0.237), which was also not statistically significant (Table 2). These results suggest that there were no meaningful differences in the effect of light therapy across these participant types, indicating that the effect of light therapy on depressive symptoms was not significantly influenced by participant type.

Light delivery methods. The meta-regression analysis examining the effect of light delivery methods on the overall effect size of light therapy found no significant difference between the Light box and Other lighting devices. The estimate for Other lighting devices was −0.175 (*z* = −0.543, *p* = 0.587), indicating no statistically significant difference in effect size between light box and other methods of light delivery (Table 2).

Light spectrum types. The meta-regression analysis assessing the effect of light spectrum types on the overall effect size of light therapy showed varied results when comparing the reference group (Full spectrum light) to other spectrum types. The analysis revealed that bluish light had an estimate of 0.460 (z = 0.217, *p* = 0.224), indicating no statistically significant difference in effect size compared to full spectrum light. In contrast, white light exhibited a statistically significant positive effect with an estimate of 0.732 (z = 2.489, *p* = 0.013), suggesting that white light was associated with a larger effect size compared to full spectrum light. Meanwhile, green light had an estimate of 0.336 (z = 0.635, *p* = 0.526), which was not statistically significant, indicating no meaningful difference from the full spectrum light group (Table 2).

The subgroup analysis based on light spectrum types revealed notable differences in effect sizes. The full-spectrum light group had an effect size of 0.00 (95% CI: −0.72, 0.71), indicating no significant impact on depressive symptoms. In contrast, the bluish light group showed a moderate positive effect (0.51, 95% CI: 0.28, 0.74), while the white light group exhibited a stronger effect (0.74, 95% CI: 0.45, 1.02). The green light group had a smaller, non-significant effect size of 0.34 (95% CI: −0.14, 0.83). These findings suggest that bluish and white light were the most effective, while full-spectrum and green light had minimal or non-significant effects on depressive symptoms (Figure S1).

**Figure 3:**
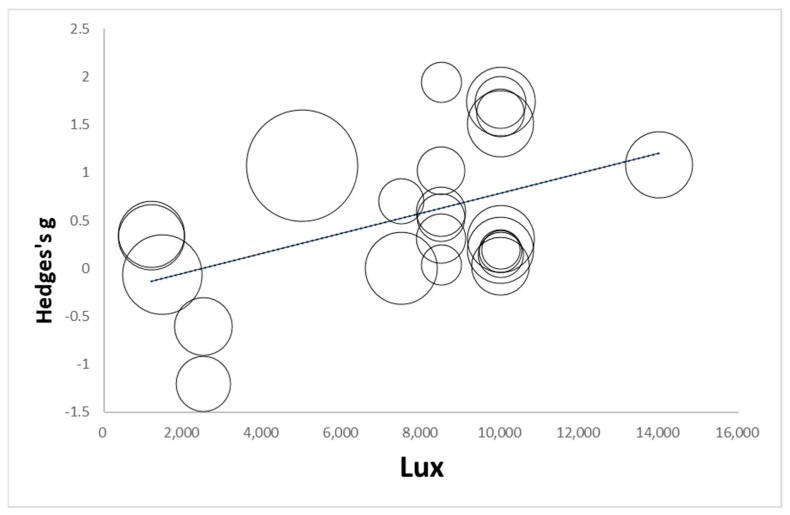
Meta-regression of the effects of light intensity (Lux).

Light intensity. To analyze the impact of light intensity on depressive symptoms, we examined the relationship between light intensity and the overall effect size of light therapy. To reduce variability in light delivery methods, we limited the analysis to studies that used light box devices. The analysis showed that light intensity had a statistically significant positive effect on the overall effect size (Estimate = 9.851 × 10^−5^, SE = 3.887 × 10^−5^, z = 2.534, *p* = 0.011) (Table 2). This suggests that as light intensity increases, the therapeutic effect of light therapy on depressive symptoms becomes more substantial. In other words, higher light intensities were associated with greater improvement in depressive symptoms. Figure 3 presents a meta-regression of the relationship between light intensity (Lux) and effect size (Hedges’ g). Each circle represents a study, with the size of the circle indicating the study’s weight in the analysis. The x-axis represents light intensity in Lux, and the y-axis shows the effect size, measured as Hedges’ g. The regression line in Figure 3 shows a slight upward trend, suggesting a potential positive association between light intensity and effect size. Smaller circles represent studies with relatively lower weights (often smaller sample sizes), while larger circles indicate studies with higher weights. Most data points are symmetrically distributed around the regression line, suggesting that higher light intensity may be associated with a slightly increased effect size. In conclusion, the meta-regression suggests a positive trend, indicating that increased light intensity is associated with a greater therapeutic effect on depressive symptoms.

The results of the subgroup analysis based on light intensity reveal varying effect sizes. For the group exposed to below 5000 lux, the pooled effect size was −0.01 (95% CI: −0.64, 0.62), indicating no significant effect. The group exposed to 5000 to less than 10,000 lux showed a moderate effect with a pooled effect size of 0.62 (95% CI: 0.21, 1.02). Finally, the group exposed to 10,000 lux and above demonstrated a larger effect size of 0.74 (95% CI: 0.32, 1.16), suggesting a stronger impact on depressive symptoms. In summary, the findings indicate that higher light intensities (10,000 lux and above) are associated with a more significant positive effect on depressive symptoms, while lower intensities (below 5000 lux) showed minimal or no effect (Figure S2).

Length of light exposure. A meta-regression analysis was conducted to examine the impact of light exposure duration per session on the overall effect size of light therapy. The analysis revealed that light exposure duration did not have a significant effect on the effect size (Estimate = −1.973 × 10^−4^, SE = 8.998 × 10^−4^, z = −0.219, *p* = 0.826) (Table 2). This suggests that increasing the duration of light exposure per session does not lead to a significant change in the therapeutic effect on depressive symptoms.

Light therapy timing. The meta-regression analysis investigating the impact of light therapy timing on the overall effect size yielded the following results. The reference group, which received light therapy in the morning, was compared to groups receiving therapy at different times of the day: midday/afternoon, evening, and other times (i.e., morning and evening or just evening). The results showed no statistically significant differences in the overall effect size between the groups. For the midday group, the estimate was −0.273 (z = −0.819, *p* = 0.413), indicating no significant difference compared to the morning group. The evening group had an estimate of 0.844 (z = 1.602, *p* = 0.109), suggesting a non-significant positive trend in effect size. Similarly, the other times group had an estimate of 0.345 (z = 1.278, *p* = 0.201), also showing no significant difference (Table 2).

Intervention duration. The meta-regression analysis examining the impact of total intervention duration on the overall effect size revealed no statistically significant association. The estimate for the intervention period was −7.587 × 10^−4^ (SE = 0.001, z = −0.511, *p* = 0.609), indicating that the length of the total intervention period did not significantly influence the effect size (Table 2).

Control type. The meta-regression analysis assessing the impact of control type on the overall effect size of light therapy showed significant results. The reference group, which received a sham intervention (i.e., placebo light exposure), was compared to a control group that did not receive any light exposure (i.e., no treatment or treatment as usual). The results indicated a statistically significant difference between the two groups. Specifically, the non-light exposure group had a significantly higher effect size compared to the sham intervention group, with an estimate of 0.567 (z = 2.360, *p* = 0.018) (Table 2). This suggests that studies using a non-light exposure control group tend to report larger effect sizes than those using a sham light intervention.

The results of the subgroup analysis based on control type reveal notable differences in effect sizes. The group with no light exposure demonstrated a pooled effect size of 0.13 (95% CI: −0.28, 0.54), indicating no significant effect. In contrast, the group using placebo (i.e., dim light exposure) showed a much larger pooled effect size of 0.70 (95% CI: 0.44, 0.95), suggesting a significant positive effect. These results indicate that studies using passive controls tend to show larger positive effects of light therapy on depressive symptoms, while those using active controls (placebo light) showed minimal or no effect (Figure S3).

### 3.6. Analysis of Publication Bias

**Figure 4:**
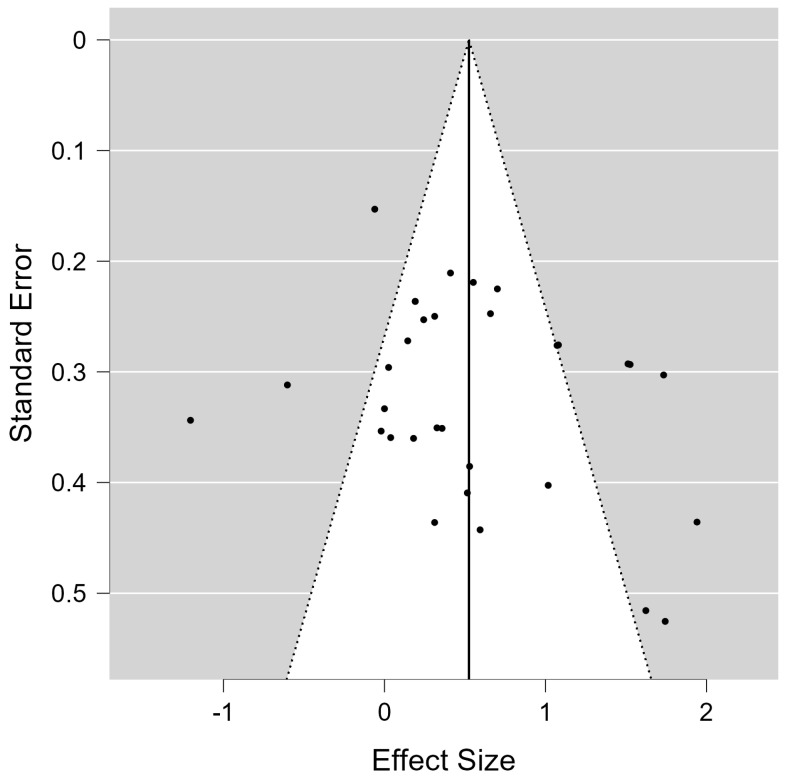
The funnel plot of included studies.

Publication bias was assessed using multiple methods, including Kendall’s τ rank correlation test and Egger’s regression test for funnel plot asymmetry [36,68]. Figure 4 presents the funnel plot of the included studies, a visual tool for evaluating potential publication bias. In this plot, each study’s effect size is on the x-axis, and the standard error is on the y-axis. In the absence of publication bias, studies should symmetrically cluster around the pooled effect size (center line) within the triangular region representing the 95% confidence limits. Upon inspecting Figure 4, a relatively symmetrical distribution is observed around the central line, with most studies falling within the expected funnel region. This distribution suggests that both smaller studies (with larger standard errors, near the bottom) and larger studies (with smaller standard errors, near the top) are evenly spread around the mean effect size, implying minimal publication bias. The Kendall’s τ rank test yielded a correlation of 0.165 (*p* = 0.191), indicating no significant asymmetry in the funnel plot and thus no strong evidence of publication bias. Similarly, Egger’s regression test for asymmetry showed a z-value of 1.597 (*p* = 0.110), further supporting the absence of substantial publication bias.

To further confirm these findings, Rosenthal’s fail-safe N was calculated, yielding a value of 1021. This high number indicates that 1021 additional studies with null results would be required to negate the significance of the observed effects, providing further evidence for the robustness of the results. The trim and fill method for assessing and correcting publication bias also showed that no additional studies were added, suggesting that no correction for funnel plot asymmetry is necessary. In summary, the visual symmetry in Figure 4 and the results from Kendall’s τ, Egger’s test, Rosenthal’s fail-safe N, and the trim and fill method all indicate that publication bias is minimal or absent in this meta-analysis. These findings strengthen the credibility of the observed effect sizes in the included studies.

## 4. Discussion

The purpose of this meta-analysis was to evaluate the effectiveness of light therapy on reducing depressive symptoms in elderly adults. The results demonstrated that light therapy had a statistically significant effect on alleviating depressive symptoms, with an overall effect size of 0.525 (95% CI: 0.292–0.758, *p* < 0.001). This indicates a medium effect size, suggesting that light therapy may provide meaningful improvements in depressive symptoms for older adults. Additionally, no significant publication bias was detected, as confirmed by the funnel plot and statistical tests. The trim and fill analysis results indicated that no additional studies were needed to adjust for missing studies, reinforcing the robustness of our findings. The results of the meta-regression and subgroup analyses revealed that both light intensity and light spectrum were significant factors influencing the overall effect size of light therapy on depressive symptoms.

Meta-regression analysis showed that higher light intensity (e.g., 10,000 lux and above) had a significant positive impact on reducing depressive symptoms. Moreover, light spectrum was identified as another key factor. The analysis indicated that white light and bluish light were the most effective in alleviating depressive symptoms, with white light showing the strongest effect size compared to other spectrums. However, factors such as the total duration of the intervention and the session length did not show a significant impact on the overall effect size. Additionally, the time of day when the light therapy was administered (morning, midday, or evening) did not result in statistically significant differences in outcomes. These findings suggest that light intensity and light spectrum are crucial factors in determining the efficacy of light therapy for reducing depressive symptoms, whereas the timing and length of exposure may have less influence.

Similar to previous meta-analyses, our study found that light therapy had a moderate effect size in reducing depressive symptoms among older adults [22,29,30]. Tao et al. (2020) [22] conducted a meta-analysis to assess the effectiveness of light therapy for non-seasonal depression. The results showed that light therapy had a mild to moderate effect in improving depressive symptoms, with an overall effect size of −0.405 (95% CI: −0.597, −0.212, *p* < 0.001). However, of the 23 studies included, only three specifically targeted elderly individuals, while the remaining 20 focused on adults under the age of 60. Additionally, the meta-analysis focusing on elderly populations found that light therapy has a moderate effect size in alleviating depressive symptoms among older adults (effect size: −0.405, 95% CI: −0.597, −0.212, *p* < 0.001), which is comparable to the effectiveness observed in studies involving other adult populations [29]. Another meta-analysis focused on elderly populations, conducted by Aini et al. (2024) [30], also reported a moderate effect size for light therapy on depressive symptoms (Hedges’ g = −0.460, 95% CI: −0.720, −0.200, *p* < 0.001). While previous meta-analyses included fewer than 10 trials and less recent data, our study incorporates up-to-date data (through 2024) and a larger dataset, with 22 clinical trials, thereby enhancing the reliability of our findings [29,30]. These results further support light therapy as an effective non-pharmacological intervention for the elderly.

Additionally, our analysis examined various light intensities and spectra, allowing for a more in-depth assessment of how different aspects of light therapy impact depressive symptoms in older adults. Among these aspects, light intensity emerged as a particularly influential factor in treatment outcomes. Specifically, studies with higher light intensities (10,000 lux and above) demonstrated stronger effects on depressive symptoms compared to lower intensities (Figure S2). Previous research supports this, showing that higher light intensity is linked to greater improvements in depressive symptoms [20,69,70,71,72]. For instance, Rosenthal’s 1984 study used 2500 lux for 3 h sessions, which initially demonstrated efficacy [73]. However, subsequent studies revealed that 10,000 lux administered for 30–40 min achieved remission rates of approximately 75%, surpassing the results of lower intensities like 3000 lux, which were significantly less effective [74,75]. A review on the mechanisms of light therapy for depression suggests that bright light modulates the autonomic nervous system and circadian rhythms, which may be crucial to its antidepressant effects [20,25,26]. Bright light, especially at higher intensities, stimulates the suprachiasmatic nucleus (SCN), regulating sleep-wake cycles and mood-related physiological processes [20,26]. Thus, 10,000 lux has become the standard recommendation for light therapy in depression. Although lower intensities can still produce effects, they require significantly longer exposure times. This underscores the importance of optimizing light intensity, especially in elderly populations who may respond differently due to age-related circadian changes and decreased light perception. 

The light spectrum type also played a crucial role in moderating the effects of light therapy on depressive symptoms. The meta-regression and subgroup analyses of light spectrum types revealed that white light had the most substantial positive effect on depressive symptoms, with a significant effect size of 0.74. Bluish light also showed a moderate positive effect (0.51), whereas full-spectrum and green light exhibited minimal or non-significant effects. These findings suggest that white light is the most effective in alleviating depressive symptoms compared to other light spectrum types. White light was utilized as the primary light source in 11 out of the 22 studies. The reason for the greater efficacy of white light may be linked to its broader spectral composition including blue and green light wavelengths [76,77]. White light covers a wide range of wavelengths, stimulating a broader range of photoreceptors in the eyes, including melanopsin-containing retinal ganglion cells [78,79]. These cells are essential in regulating the circadian rhythms through their connection to the suprachiasmatic nucleus (SCN), the body’s internal clock, which plays a pivotal role in mood regulation [25,26,80].

Furthermore, white light therapy has been shown to promote a stronger alignment of the circadian rhythm with environmental light-dark cycles, thereby enhancing sleep quality and stabilizing mood [81]. This broader spectral range of white light makes it more effective in addressing the circadian disruptions that often contribute to depressive symptoms, especially in older adults, who frequently experience misaligned circadian rhythms due to aging [82]. These findings align with previous studies, which have shown that different light spectra can have varying impacts on mood and circadian regulation [21,29,76]. Future research should focus on investigating how specific light spectra can be optimized to maximize therapeutic outcomes, particularly for non-seasonal depression in older adults, where circadian rhythm disturbances are common. 

In addition to the above findings, the meta-regression revealed that timing of light therapy did not significantly influence the treatment effect, with no meaningful differences observed between morning, midday, or evening sessions. This suggests that the time of day may not be a critical factor in determining the success of light therapy for depression in older adults. However, further research is needed to confirm this, particularly since circadian rhythm regulation is believed to be one of the mechanisms by which light therapy exerts its antidepressant effects.

Our study offers several advantages compared to previous meta-analyses. This meta-analysis focused on the elderly population, enabling us to evaluate treatment effectiveness tailored to their specific physical and psychological needs. Additionally, we reduced heterogeneity across studies, leading to more robust and consistent results for elderly depression treatment. Notably, this study included a substantially larger dataset, incorporating 22 clinical trials and 1290 participants, compared to prior meta-analyses such as Chang’s, which only included 8 trials and 426 participants [29]. As a result, the conclusions drawn from this study may offer greater reliability. Furthermore, we used meta-regression to assess the impact of various light therapy protocols and clinical variables on elderly depression, which could not be done in earlier studies due to the limited number of trials. Given that older adults are more susceptible to adverse effects from pharmacotherapy and often have limited access to psychotherapy, our meta-regression provides critical insights into the modulators of light therapy efficacy. This contributes to the development of more individualized, evidence-based treatment strategies for this vulnerable population.

There are several limitations to consider in this meta-analysis. First, the heterogeneity observed across studies highlights the variability in study design and methodology, which may affect the generalizability of the findings. Second, while we included studies with both placebo and no-treatment controls, the difference in effect sizes between these control types suggests that placebo effects may be contributing to the outcomes in some cases. This underscores the importance of carefully selecting control conditions in future studies to ensure that the effects of light therapy are not overstated. Third, although we found that light intensity plays a key role in treatment efficacy, other factors such as the duration of light exposure and total intervention period did not significantly influence outcomes. Future research should further investigate these factors, particularly to identify the optimal duration and period for light therapy in elderly populations. Fourth, the presence of publication bias, although not found to be significant, should be acknowledged. While our analysis showed minimal evidence of bias, the relatively small number of studies included in certain subgroups, such as those using specific light spectra or intensities, suggests that more research is needed to fully understand the range of effects that light therapy can offer. Fifth, one limitation of this review is the inability to specify the exact dates of the Google Scholar searches. Although searches were conducted between December 2023 and January 2024, and again between June and July 2024, the precise dates were not recorded. This limitation may affect the reproducibility of the search results, as Google Scholar’s dynamic indexing can yield different results depending on the exact search date. Future studies should consider recording specific search dates to enhance reproducibility. Lastly, this meta-analysis faces limitations in analyzing effects by gender, as most included studies did not provide means and standard deviations for gender. Consequently, subgroup analyses based on gender were not feasible, restricting the evaluation of gender-specific outcomes. Future research should prioritize the systematic collection of gender-related data to address this limitation.

In conclusion, this meta-analysis demonstrates that light therapy is an effective non-pharmacological intervention for reducing depressive symptoms in older adults, especially in studies utilizing higher light intensities. Compared to previous meta-analyses, our study incorporates more recent data and a larger number of clinical trials, providing a stronger basis for its findings and enabling a more nuanced analysis of light therapy parameters. The findings suggest that light therapy can serve a viable alternative to traditional treatments such as antidepressants and psychotherapy, particularly for elderly patients who may encounter difficulties with these options. However, given the variability in study designs and outcomes, more research is needed to refine the parameters of light therapy—such as intensity, duration, and light spectrum type—to optimize its therapeutic potential. Additionally, future studies should address the potential for placebo effects by carefully designing control conditions that accurately reflect the true efficacy of light therapy. Despite these limitations, the current findings provide valuable evidence for the clinical application of light therapy in treating depressive symptoms among elderly individuals and highlight the need for further research to better understand its full potential.

## Figures and Tables

**Table 1:** Study characteristics of 22 studies selected for the meta-analysis.

Author & Year	Diagnosis	Case Number	Age	Intervention Type *	Intervention Protocol **	Control Type	Measurement Tool
Burns et al., 2009 [47]	Dementia	T: Late morning intervention (*n* = 21, 29% male), C: Placebo (n = 25, 40% male)	T: 84.5 (1.7), C: 82.5 (1.5)	Intensity: 10,000 Device: Light box Light type: Full-spectrum light	Duration: 14 Frequency: 1 Length of therapy: 120	Standard fluorescent tube light	Cornell Scale for Depression in Dementia
Dowling et al., 2007 [48]	Alzheimer’s disease	T1: Morning intervention (*n* = 29, 24% male), T2: Afternoon intervention (*n* = 24, 17% male), C: Placebo (*n* = 17, 12% male)	84 (10)	Intensity: 2500 Device: Light box Light type: Full-spectrum light	Duration: 70 Frequency: 1 Length of therapy: 60	Usual indoor light exposure	Neuropsychiatric Inventory
Figueiro et al., 2019 [49]	Alzheimer’s disease and related dementia	T: Day time intervention (*n* = 46), C: Placebo (*n* = 33)	85.1 (7.1)	Intensity: 350–750 Device: Custom lighting (custom-built floor luminaires, light-boxes, and light tables) Light type: Bluish-white light	Duration: 28 Frequency: 1 Length of therapy: 540	Warm light	Cornell Scale for Depression in Dementia
Kolberg et al., 2021 [50]	Dementia	T: Midday intervention (*n* = 33, 24.2% male), C: Placebo (*n* = 36, 38.9% male)	T: 86 as the median age C: 82.5 as the median age	Intensity: 1000 Device: Ceiling-mounted LED panels Light type: Bluish light	Duration: 56 Frequency: 1 Length of therapy: 300	Warm light	Cornell Scale for Depression in Dementia
Konis et al., 2018 [51]	Alzheimer’s disease and related dementia	T: Morning intervention (*n* = 36, 32.6% male), C: Placebo (*n* = 28, 19.4% male)	T: 85.6 (7.0), C: 84.7 (7.0)	Intensity: 159.3 melanopic lux Device: Daylit room Light type: Full-spectrum light	Duration: 84 Frequency: 1 Length of therapy: 120	Typical indoor electrical lighting	Cornell Scale for Depression in Dementia
Lievers et al., 2011 [24]	Major depressive disorder	T: Early morning intervention (*n* = 40, 36% male), C: Placebo (*n* = 44, 33% male)	T: 69.7 (8.5), C: 69.0 (6.6)	Intensity: 7500 Device: Light box Light type: Bright pale blue light	Duration: 21 Frequency: 1 Length of therapy: 60	Dim red light	Hamilton Scale for Depression
Loving et al., 2005a [52]	Aging depressed people	T1: Morning intervention (*n* = 13), T2: Midday intervention (*n* = 13), T3: Evemimg intervention (*n* = 15), C1: Morning placebo (*n* = 15), C2: Midday placebo (*n* = 15), C3: Evemimg placebo (*n* = 16)	67.7 (5.45)	Intensity: 8500 Device: Light box Light type: Bright white light	Duration: 28 Frequency: 1 Length of therapy: 60	Dim red light	Geriatric Depression Scale, Hamilton Scale for Depression
Loving et al., 2005b [53]	Major And Minor Depressive Disorder	T: Morning intervention (*n* = 17), C: Placebo (*n* = 17)	67.7 (6.35)	Intensity: 1200 Device: Light box Light type: Bright green light	Duration: 28 Frequency: 1 Length of therapy: 60	Dim red light	Geriatric Depression Scale, Hamilton Scale for Depression
Onega et al., 2016 [54]	Dementia	T: Day time intervention (*n* = 30), C: Placebo (*n* = 30)	82.6 (9.6)	Intensity: 10,000 Device: Light box Light type: Bright white light	Duration: 40 Frequency: 1 Length of therapy: 60	Dim light	Cornell Scale for Depression in Dementia, Depressive Symptom Assessment for Older Adults
Onega et al., 2018 [55]	Dementia	T: Day time intervention (*n* = 30), C: Placebo (*n* = 30)	-	Device: Light box Light type: Full-spectrum light	Duration: 40	Dim light	Cornell Scale for Depression in Dementia
Paus et al., 2007 [56]	Parkinson’s disease	T: Morning intervention (*n* = 18, 66.67% male), C: Placebo (*n* = 18, 61.11% male)	T: 63.6 (9.8), C: 63.4 (9.7)	Intensity: 7500 Device: Light box Light type: Bright white light	Duration: 15 Frequency: 1 Length of therapy: 30	Dim light	Beck’s Depression Inventory
Raymackers et al., 2019 [57]	Parkinson’s disease	T: Morning intervention (*n* = 16, 62.5% male), C: Placebo (*n* = 16, 62.5% male)	-	Intensity: 472.7 Device: Head-mounted device Light type: Blue-enriched light	Duration: 30 Frequency: 1 Length of therapy: 45	Orange-enriched light	Hospital Anxiety and Depression Scale
Riemersma et al., 2008 [58]	Dementia	T: All day intervention (*n* = 47, 74% male), C: Placebo (*n* = 40, 76% male)	85.7 (5.5)	Intensity: 1000 Device: Ceiling-mounted fixtures Light type: White light	Duration: Average of 15 months Frequency: 1 Length of therapy: 540	Dim light	Cornell Scale for Depression in Dementia
Rutten et al., 2016 [59]	Parkinson’s disease and major depressive disorder	T: Morning and evening intervention (*n* = 35, 85% male), C: Placebo (*n* = 37, 83% male)	T: 58.9 (8.5), C: 65.8 (8.6)	Intensity: 10,000 Device: Light box Light type: Bright white light	Duration: 90 Frequency: 1 Length of therapy: 60	Dim light	Hamilton Depression Rating Scale, Geriatric Depression Scale
Sumaya et al., 2001 [60]	Score of 11–20 on GDS	T: Morning intervention (*n* = 10), C1: Placebo (*n* = 10) C2: no treatment (*n* = 10)	83.8 (9.56)	Intensity: 10,000 Device: Light box Light type: Bright white light	Duration: 5 Frequency: 1 Length of therapy: 30	No treatment	Geriatric Depression Scale
Tsai et al., 2004 [61]	Depressed elders	T: Morning intervention (*n* = 30, 60% male), C: no light treatment (*n* = 30, 50% male)	T: 75.3 (7.4), C: 74.6 (5.7)	Intensity: 5000 Device: Light box Light type: Bright white light	Duration: 5 Frequency: 1 Length of therapy: 50	Treatment as usual	Geriatric Depression Scale
Videnovic et al., 2017 [62]	Parkinson’s disease	T: Day time intervention (*n* = 16, 50% male), C: Placebo (*n* = 15, 33.33% male)	T: 62.31 (10.83), C: 64.07 (8.89)	Intensity: 10,000 Device: Light box Light type: Bright white light	Duration: 14 Frequency: 1 Length of therapy: 120	Dim red light	Beck Depression Inventory
Wu et al., 2015 [63]	Depressed elders	T: Morning intervention (*n* = 34, 52.9% male), C: no light treatment (*n* = 31, 61.3% male)	T: 80.97 (9.84), C: 79.03 (10.06)	Intensity: 10,000 Device: Light box Light type: Bright white light	Duration: 12 Frequency: 1 Length of therapy: 30	Treatment as usual	Geriatric Depression Scale—Short Form
Ouslander et al., 2006 [64]	Nursing Home Patients	T: Early morning intervention (*n* = 77, 17% male), C: no light treatment (*n* = 96, 33% male)	T: 83.5 (8.71), C: 82.9 (9.3)	Intensity: 10,000 Device: Light box Light type: Bright white light	Duration: 17 Frequency: 1 Length of therapy: 120	No treatment	Geriatric Depression Scale
Kim et al., 2021 [65]	Alzheimer’s disease	T: Morning intervention (*n* = 14, 85.71% male), C: Placebo (*n* = 11, 45.45% male)	T: 77.36 (5.79), C: 78.55 (7.71)	Intensity: 30 lux at the eyes Device: Light box Light type: Blue-enriched white light	Duration: 14 Frequency: 1 Length of therapy: 60	Dim light	Cornell Scale for Depression in Dementia
Livingstone et al., 2019 [66]	Dementia	T: Morning intervention (*n* = 42, 21% male), C: no light treatment (*n* = 20, 50% male)	T: 80.4 (9), C: 79.6 (7)	Intensity: 10,000 Device: Light box Light type: Bright white light	Duration: 43–64 Frequency: 1 Length of therapy: 30	Treatment as usual	Hospital Anxiety and Depression Scale
Zou et al., 2022 [67]	Alzheimer’s disease-related dementia	T: Morning intervention (*n* = 34, 47.06% male), C: Placebo (*n* = 27, 70.59% male)	T: 75.94 (9.47), C: 73.04 (9.34)	Intensity: 14,000 Device: High-intensity light therapy device	Duration: 28 Frequency: 1 Length of therapy: 30	Dim light	Neuropsychiatric Inventory

Abbreviation: C, Control; T, Treatment; TAU, Treatment as usual. * The unit of intensity for light is lux. ** The duration is the amount of light exposure time measured in minutes per day. The frequency refers to how many times the intervention is administered per day. The unit of time for therapy sessions is the minute.

**Table 2:** Meta-regression analysis of light therapy studies on depression symptom.

Predictor Variables	Estimates	SE	Z	*P*	95% Confidence Interval	
					Lower	Upper
Particiapnt type						
Depression	0.253	0.262	0.965	0.334	−0.260	0.766
Parkinson	−0.404	0.342	−1.181	0.237	−1.074	0.266
Light delivery methods						
Others	−0.175	0.321	−0.543	0.587	−0.804	0.455
Light spectrum types						
Blue	0.460	0.378	1.217	0.224	−0.281	1.200
White	0.732	0.294	2.489	0.013	0.156	1.309
Green	0.336	0.529	0.635	0.526	−0.701	1.372
Light intensity	9.851 × 10^−5^	3.887 × 10^−5^	2.534	0.011	2.232 × 10^−5^	1.747 × 10^−5^
Length of light exposure	−1.973 × 10^−4^	8.998 × 10^−4^	−0.219	0.826	−0.002	0.002
Light therpy timing						
Midday	−0.273	0.333	−0.819	0.413	−0.926	0.380
Evening	0.844	0.527	1.602	0.109	−0.189	1.877
Others	0.345	0.270	1.278	0.201	−0.184	0.875
Intervention duration	−7.587 × 10^−4^	0.001	−0.511	0.609	−0.004	0.002
Control type						
Others	0.567	0.240	2.360	0.018	0.096	1.038

## Data Availability

The data supporting the findings of this study are available from the corresponding author upon reasonable request.

## Supplementary Materials

The following supporting information can be downloaded at: https://www.mdpi.com/article/10.3390/jcm13226982/s1, Figure S1. The results of subgroup analysis based on light spectrum types; Figure S2. The results of subgroup analysis based on light intensity; Figure S3. The results of subgroup analysis based on control types; Table S1. Risk of bias; PRISMA checklist.

## References

[1] Hu T., Zhao X., Wu M., Li Z., Luo L., Yang C., Yang F. (2022). Prevalence of depression in older adults: A systematic review and meta-analysis. Psychiatry Res..

[2] Herrman H., Patel V., Kieling C., Berk M., Buchweitz C., Cuijpers P., Furukawa T.A., Kessler R.C., Kohrt B.A., Maj M. (2022). Time for united action on depression: A Lancet–World Psychiatric Association Commission. Lancet.

[3] Liao D.-D., Dong M., Ding K.-R., Hou C.-L., Tan W.-Y., Ke Y.-F., Jia F.-J., Wang S.-B. (2023). Prevalence and patterns of major depressive disorder and subthreshold depressive symptoms in south China. J. Affect. Disord..

[4] Abdoli N., Salari N., Darvishi N., Jafarpour S., Solaymani M., Mohammadi M., Shohaimi S. (2022). The global prevalence of major depressive disorder (MDD) among the elderly: A systematic review and meta-analysis. Neurosci. Biobehav. Rev..

[5] Zenebe Y., Akele B., W/Selassie M., Necho M. (2021). Prevalence and determinants of depression among old age: A systematic review and meta-analysis. Ann. Gen. Psychiatry.

[6] Jafari-Koulaee A., Mohammadi E., Fox M.T., Rasekhi A., Akha O. (2024). Predictors of basic and instrumental activities of daily living among older adults with multiple chronic conditions. BMC Geriatr..

[7] Liu H., Ma Y., Lin L., Sun Z., Li Z., Jiang X. (2023). Association between activities of daily living and depressive symptoms among older adults in China: Evidence from the CHARLS. Front. Public Health.

[8] Botoseneanu A., Elman M.R., Allore H.G., Dorr D.A., Newsom J.T., Nagel C.L., Quiñones A.R. (2023). Depressive multimorbidity and trajectories of functional status among older Americans: Differences by racial/ethnic group. J. Am. Med. Dir. Assoc..

[9] Giannouli V., Tsolaki M. (2023). Beneath the Top of the Iceberg: Financial Capacity Deficits in Mixed Dementia with and without Depression. Healthcare.

[10] Parajuli J., Berish D., Jao Y.-L. (2021). Chronic conditions and depressive symptoms in older adults: The mediating role of functional limitations. Aging Ment. Health.

[11] Hybels C.F., Pieper C.F., Blazer D.G. (2009). The complex relationship between depressive symptoms and functional limitations in community-dwelling older adults: The impact of subthreshold depression. Psychol. Med..

[12] Ni Z., Zhu X., Tian K., Chen Q., Yang Y., Xie S. (2024). Depressive symptoms of older adults with chronic diseases: The mediating roles of activities of daily living and economic burden of diseases. Front. Psychol..

[13] Hoertel N., Rotenberg L., Schuster J.-P., Blanco C., Lavaud P., Hanon C., Hozer F., Teruel E., Manetti A., Costemale-Lacoste J.-F. (2021). Generalizability of pharmacologic and psychotherapy trial results for late-life unipolar depression. Aging Ment. Health.

[14] Ainsworth N.J., Marawi T., Maslej M.M., Blumberger D.M., McAndrews M.P., Perivolaris A., Pollock B.G., Rajji T.K., Mulsant B.H. (2024). Cognitive outcomes after antidepressant pharmacotherapy for late-life depression: A systematic review and meta-analysis. Am. J. Psychiatry.

[15] Romdhani A., Lehmann S., Schlatter J. (2023). Discontinuation of Antidepressants in Older Adults: A Literature Review. Ther. Clin. Risk Manag..

[16] Baba H. (2023). Treatment strategy for late-life depression. Psychiatry Clin. Neurosci. Rep..

[17] Dong C., Shi H., Liu P., Si G., Yan Z. (2022). A critical overview of systematic reviews and meta-analyses of light therapy for non-seasonal depression. Psychiatry Res..

[18] Pail G., Huf W., Pjrek E., Winkler D., Willeit M., Praschak-Rieder N., Kasper S. (2011). Bright-light therapy in the treatment of mood disorders. Neuropsychobiology.

[19] Terman M. (2007). Evolving applications of light therapy. Sleep Med. Rev..

[20] Oldham M.A., Ciraulo D.A. (2014). Bright light therapy for depression: A review of its effects on chronobiology and the autonomic nervous system. Chronobiol. Int..

[21] Blume C., Garbazza C., Spitschan M. (2019). Effects of light on human circadian rhythms, sleep and mood. Somnologie.

[22] Tao L., Jiang R., Zhang K., Qian Z., Chen P., Lv Y., Yao Y. (2020). Light therapy in non-seasonal depression: An update meta-analysis. Psychiatry Res..

[23] Do A., Li V.W., Huang S., Michalak E.E., Tam E.M., Chakrabarty T., Yatham L.N., Lam R.W. (2022). Blue-light therapy for seasonal and non-seasonal depression: A systematic review and meta-analysis of randomized controlled trials. Can. J. Psychiatry.

[24] Lieverse R., Van Someren E.J., Nielen M.M., Uitdehaag B.M., Smit J.H., Hoogendijk W.J. (2011). Bright light treatment in elderly patients with nonseasonal major depressive disorder: A randomized placebo-controlled trial. Arch. Gen. Psychiatry.

[25] Bais B., Hoogendijk W.J., Lambregtse-van den Berg M.P. (2021). Light therapy for mood disorders. Handb. Clin. Neurol..

[26] Huang X., Tao Q., Ren C. (2024). A comprehensive overview of the neural mechanisms of light therapy. Neurosci. Bull..

[27] Terman M., Terman J.S. (2005). Light therapy for seasonal and nonseasonal depression: Efficacy, protocol, safety, and side effects. CNS Spectr..

[28] Maruani J., Geoffroy P.A. (2019). Bright light as a personalized precision treatment of mood disorders. Front. Psychiatry.

[29] Chang C.-H., Liu C.-Y., Chen S.-J., Tsai H.-C. (2018). Efficacy of light therapy on nonseasonal depression among elderly adults: A systematic review and meta-analysis. Neuropsychiatr. Dis. Treat..

[30] Aini N., Chen R., Chu H., Chang C.-Y., Lin H.-C., Jen H.-J., Liu D., Lee T.-Y., Chou K.-R. (2024). The effects of light therapy on sleep, depression, neuropsychiatric Behaviors, and cognition among people living with dementia: A meta-analysis of randomized controlled trials. Am. J. Geriatr. Psychiatry.

[31] Page M.J., McKenzie J.E., Bossuyt P.M., Boutron I., Hoffmann T.C., Mulrow C.D., Shamseer L., Tetzlaff J.M., Akl E.A., Brennan S.E. (2021). The PRISMA 2020 statement: An updated guideline for reporting systematic reviews. BMJ.

[32] PROSPERO International Prospective Register of Systematic Reviews. https://www.crd.york.ac.uk/prospero/.

[33] Cumpston M., Li T., Page M.J., Chandler J., Welch V.A., Higgins J.P., Thomas J. (2019). Updated guidance for trusted systematic reviews: A new edition of the Cochrane Handbook for Systematic Reviews of Interventions. Cochrane Database Syst. Rev..

[34] Schwarzer G. (2022). Meta-analysis in R. Systematic Reviews in Health Research: Meta-Analysis in Context.

[35] Schwarzer G., Carpenter J.R., Rücker G. (2015). Meta-Analysis with R.

[36] Borenstein M., Hedges L.V., Higgins J.P., Rothstein H.R. (2021). Introduction to Meta-Analysis.

[37] Hedges L.V., Olkin I. (2014). Statistical Methods for Meta-Analysis.

[38] Brydges C.R. (2019). Effect size guidelines, sample size calculations, and statistical power in gerontology. Innov. Aging.

[39] Cohen J. (2013). Statistical Power Analysis for the Behavioral Sciences.

[40] Lin L. (2020). Comparison of four heterogeneity measures for meta-analysis. J. Eval. Clin. Pract..

[41] Higgins J.P., López-López J.A., Aloe A.M. (2020). Meta-regression. Handbook of Meta-Analysis.

[42] Cinar O., Umbanhowar J., Hoeksema J.D., Viechtbauer W. (2021). Using information-theoretic approaches for model selection in meta-analysis. Res. Synth. Methods.

[43] Viechtbauer W. (2020). Model checking in meta-analysis. Handbook of Meta-Analysis.

[44] Kossmeier M., Tran U.S., Voracek M. (2020). Power-enhanced funnel plots for meta-analysis. Z. Für Psychol..

[45] Nakagawa S., Lagisz M., Jennions M.D., Koricheva J., Noble D.W., Parker T.H., Sánchez-Tójar A., Yang Y., O’Dea R.E. (2022). Methods for testing publication bias in ecological and evolutionary meta-analyses. Methods Ecol. Evol..

[46] Fajar J. (2024). Approaches for identifying and managing publication bias in meta-analysis. Deka Med..

[47] Burns A., Allen H., Tomenson B., Duignan D., Byrne J. (2009). Bright light therapy for agitation in dementia: A randomized controlled trial. Int. Psychogeriatr..

[48] Dowling G.A., Graf C.L., Hubbard E.M., Luxenberg J.S. (2007). Light treatment for neuropsychiatric behaviors in Alzheimer’s disease. West. J. Nurs. Res..

[49] Figueiro M.G., Plitnick B., Roohan C., Sahin L., Kalsher M., Rea M.S. (2019). Effects of a tailored lighting intervention on sleep quality, rest–activity, mood, and behavior in older adults with Alzheimer disease and related dementias: A randomized clinical trial. J. Clin. Sleep Med..

[50] Kolberg E., Hjetland G.J., Thun E., Pallesen S., Nordhus I.H., Husebo B.S., Flo-Groeneboom E. (2021). The effects of bright light treatment on affective symptoms in people with dementia: A 24-week cluster randomized controlled trial. BMC Psychiatry.

[51] Konis K., Mack W.J., Schneider E.L. (2018). Pilot study to examine the effects of indoor daylight exposure on depression and other neuropsychiatric symptoms in people living with dementia in long-term care communities. Clin. Interv. Aging.

[52] Loving R.T., Kripke D.F., Elliott J.A., Knickerbocker N.C., Grandner M.A. (2005). Bright light treatment of depression for older adults [ISRCTN55452501]. BMC Psychiatry.

[53] Loving R.T., Kripke D.F., Knickerbocker N.C., Grandner M.A. (2005). Bright green light treatment of depression for older adults [ISRCTN69400161]. BMC Psychiatry.

[54] Onega L.L., Pierce T.W., Epperly L. (2016). Effect of bright light exposure on depression and agitation in older adults with dementia. Issues Ment. Health Nurs..

[55] Onega L.L., Pierce T.W., Epperly L. (2018). Bright light therapy to treat depression in individuals with mild/moderate or severe dementia. Issues Ment. Health Nurs..

[56] Paus S., Schmitz-Hübsch T., Wüllner U., Vogel A., Klockgether T., Abele M. (2007). Bright light therapy in Parkinson’s disease: A pilot study. Mov. Disord. Off. J. Mov. Disord. Soc..

[57] Raymackers J.-M., Andrade M., Baey E., Vanneste M., Evrard F. (2019). Bright light therapy with a head-mounted device for anxiety, depression, sleepiness and fatigue in patients with Parkinson’s disease. Acta Neurol. Belg..

[58] Riemersma-van Der Lek R.F., Swaab D.F., Twisk J., Hol E.M., Hoogendijk W.J., Van Someren E.J. (2008). Effect of bright light and melatonin on cognitive and noncognitive function in elderly residents of group care facilities: A randomized controlled trial. JAMA.

[59] Rutten S., Vriend C., Smit J.H., Berendse H.W., Hoogendoorn A.W., van den Heuvel O.A., van der Werf Y.D. (2016). A double-blind randomized controlled trial to assess the effect of bright light therapy on depression in patients with Parkinson’s disease. BMC Psychiatry.

[60] Sumaya I.C., Rienzi B.M., Deegan J.F., Moss D.E. (2001). Bright light treatment decreases depression in institutionalized older adults: A placebo-controlled crossover study. J. Gerontol. Ser. A Biol. Sci. Med. Sci..

[61] Tsai Y.F., Wong T.K., Juang Y.Y., Tsai H.H. (2004). The effects of light therapy on depressed elders. Int. J. Geriatr. Psychiatry.

[62] Videnovic A., Klerman E.B., Wang W., Marconi A., Kuhta T., Zee P.C. (2017). Timed light therapy for sleep and daytime sleepiness associated with Parkinson disease: A randomized clinical trial. JAMA Neurol..

[63] Wu M.C., Sung H.C., Lee W.L., Smith G.D. (2015). The effects of light therapy on depression and sleep disruption in older adults in a long-term care facility. Int. J. Nurs. Pract..

[64] Ouslander J.G., Connell B.R., Bliwise D.L., Endeshaw Y., Griffiths P., Schnelle J.F. (2006). A nonpharmacological intervention to improve sleep in nursing home patients: Results of a controlled clinical trial. J. Am. Geriatr. Soc..

[65] Kim S.J., Lee S.H., Suh I.B., Jang J.-W., Jhoo J.H., Lee J.H. (2021). Positive effect of timed blue-enriched white light on sleep and cognition in patients with mild and moderate Alzheimer’s disease. Sci. Rep..

[66] Livingston G., Barber J.A., Kinnunen K.M., Webster L., Kyle S.D., Cooper C., Espie C.A., Hallam B., Horsley R., Pickett J. (2019). DREAMS-START (Dementia RElAted Manual for Sleep; STrAtegies for RelaTives) for people with dementia and sleep disturbances: A single-blind feasibility and acceptability randomized controlled trial. Int. Psychogeriatr..

[67] Zou C., Mei X., Li X., Hu J., Xu T., Zheng C. (2022). Effect of light therapy on delirium in older patients with Alzheimer’s disease-related dementia. J. Psychiatr. Res..

[68] Littell J.H. (2008). Systematic Reviews and Meta-Analysis.

[69] Chiu H.L., Chan P.T., Chu H., Hsiao S.T.S., Liu D., Lin C.H., Chou K.R. (2017). Effectiveness of light therapy in cognitively impaired persons: A metaanalysis of randomized controlled trials. J. Am. Geriatr. Soc..

[70] Forbes D., Blake C.M., Thiessen E.J., Peacock S., Hawranik P. (2014). Light therapy for improving cognition, activities of daily living, sleep, challenging behaviour, and psychiatric disturbances in dementia. Cochrane Database Syst. Rev..

[71] Nussbaumer B., Kaminski-Hartenthaler A., Forneris C.A., Morgan L.C., Sonis J.H., Gaynes B.N., Greenblatt A., Wipplinger J., Lux L.J., Winkler D. (2015). Light therapy for preventing seasonal affective disorder. Cochrane Database Syst. Rev..

[72] Jiang L., Zhang S., Wang Y., So K.-F., Ren C., Tao Q. (2020). Efficacy of light therapy for a college student sample with non-seasonal subthreshold depression: An RCT study. J. Affect. Disord..

[73] Rosenthal N.E., Sack D.A., Gillin J.C., Lewy A.J., Goodwin F.K., Davenport Y., Mueller P.S., Newsome D.A., Wehr T.A. (1984). Seasonal affective disorder: A description of the syndrome and preliminary findings with light therapy. Arch. Gen. Psychiatry.

[74] Terman J.S., Terman M., Schlager D., Rafferty B., Rosofsky M., Link M., Gallin P., Quitkin F. (1990). Efficacy of brief, intense light exposure for treatment of winter depression. Psychopharmacol. Bull..

[75] Magnusson A., Kritbjarnarson H. (1991). Treatment of seasonal affective disorder with high-intensity light: A phototherapy study with an Icelandic group of patients. J. Affect. Disord..

[76] Alotaibi M.A., Halaki M., Chow C.-M. (2016). A systematic review of light therapy on mood scores in major depressive disorder: Light specification, dose, timing and delivery. Int. J. Sci. Basic Appl. Res..

[77] Roecklein K.A., Wong P.M., Miller M.A., Donofry S.D., Kamarck M.L., Brainard G.C. (2013). Melanopsin, photosensitive ganglion cells, and seasonal affective disorder. Neurosci. Biobehav. Rev..

[78] Hannibal J., Fahrenkrug J. (2004). Melanopsin containing retinal ganglion cells are light responsive from birth. Neuroreport.

[79] Feigl B., Carter D.D., Zele A.J. (2023). Photoreceptor enhanced light therapy (PELT): A framework for implementing BiologicallyDirected integrative lighting. Leukos.

[80] Berk M. (2009). Sleep and depression-theory and practice. Aust. Fam. Physician.

[81] Faulkner S.M., Dijk D.-J., Drake R.J., Bee P.E. (2020). Adherence and acceptability of light therapies to improve sleep in intrinsic circadian rhythm sleep disorders and neuropsychiatric illness: A systematic review. Sleep Health.

[82] Kripke D.F., Risch S.C., Janowsky D. (1983). Bright white light alleviates depression. Psychiatry Res..

