# Supplementary material for: Light Therapy for Older People with Depressive Symptoms: Systematic Review and Meta-Analysis
Source: J Clin Med. 2024 Nov 20;13(22):6982. doi: 10.3390/jcm13226982 (PMC11594984; doi:10.3390/jcm13226982)
Supplement: Supplementary file 1 [file jcm-13-06982-s001.zip › supplementary.pdf]

Table S1. Risk of bias.

Study	Bias due to the randomization process	Bias due to deviations from intended interventions	Bias due to missing outcome data	Bias in measurement of the outcome	Bias in selection of the reported result	RoB 2 overall bias
Burns et al., 2009	Some Concerns	Some Concerns	Low	Low	Low	Some Concerns
Dowling et al., 2007	Some Concerns	Some Concerns	Some Concerns	High	Some Concerns	High
Figueiro et al., 2019	Some Concerns	Some Concerns	Some Concerns	Low	Low	Some Concerns
Kolberg et al., 2021	Some Concerns	Some Concerns	Some Concerns	High	Low	High
Konis et al., 2018	High	Some Concerns	Some Concerns	High	Low	High
Lievers et al., 2011	Some Concerns	Some Concerns	Some Concerns	Some Concerns	Low	Some Concerns
Loving et al., 2005a	Some Concerns	Some Concerns	Low	Low	Low	Some Concerns
Loving et al., 2005b	Some Concerns	Some Concerns	Low	Low	Low	Some Concerns
Onega et al., 2016	Some Concerns	Some Concerns	Some Concerns	High	Low	High
Onega et al., 2018	Some Concerns	Some Concerns	Some Concerns	High	Low	High
Paus et al., 2007	High	Some Concerns	Low	Low	Low	High
Raymackers et al., 2019	Some Concerns	Some Concerns	Low	Some Concerns	Low	Some Concerns
Riemersma et al., 2008	Some Concerns	Some Concerns	Some Concerns	Low	Low	Some Concerns
Rutten et al., 2016	Some Concerns	Some Concerns	Low	Low	Low	Some Concerns
Sumaya et al., 2001	Some Concerns	Some Concerns	Some Concerns	Some Concerns	Low	Some Concerns
Tsai et al., 2004	Some Concerns	Some Concerns	Some Concerns	Some Concerns	Low	Some Concerns
Videnovic et al., 2017	Some Concerns	Some Concerns	Low	Low	Low	Some Concerns
Wu et al., 2015	Some Concerns	Some Concerns	Some Concerns	Some Concerns	Low	Some Concerns
Ouslander et al., 2006	Some Concerns	Some Concerns	High	Some Concerns	Low	High
Kim et al., 2021	Some Concerns	Some Concerns	Some Concerns	Some Concerns	Low	Some Concerns
Livingstone et al., 2019	Low	Some Concerns	Some Concerns	Low	Low	Some Concerns
Zou et al., 2022	Some Concerns	Some Concerns	Some Concerns	Some Concerns	Low	Some Concerns

Randomized controlled trials (RCTs) were evaluated with the Cochrane risk-of-bias tool for randomized trials (RoB 2).

a) Light spectrum types – Full spectrum

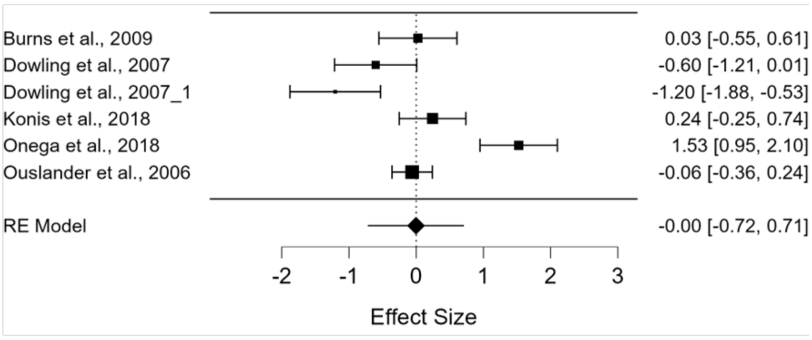

b) Light spectrum types – Blue light

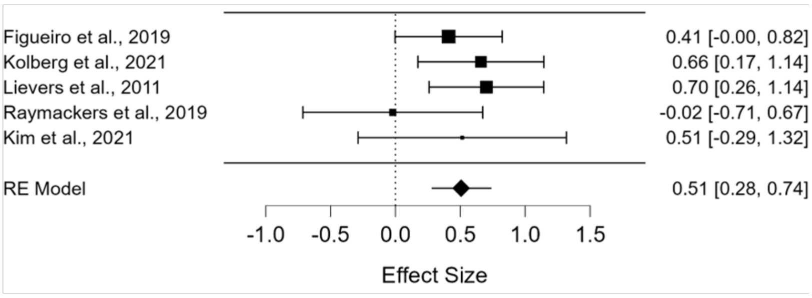

c) Light spectrum types – White light

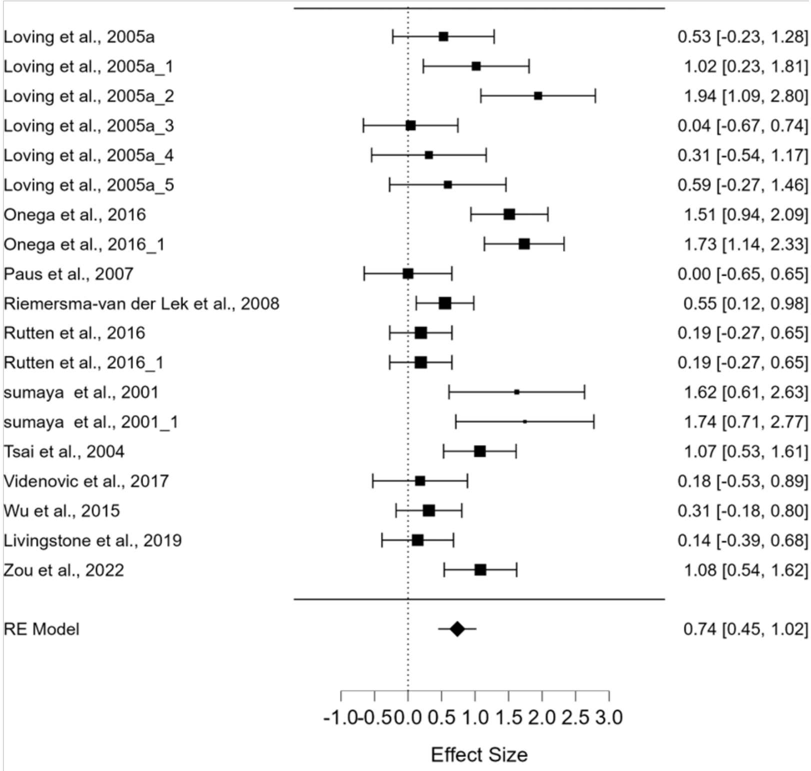

d) Light spectrum types – Green light

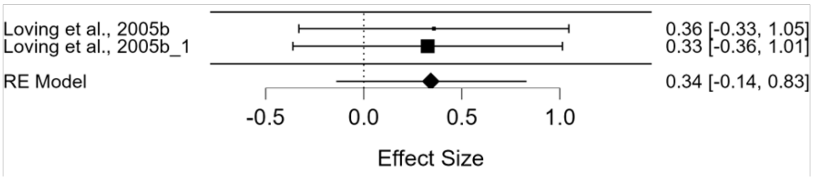

Figure S1. The results of subgroup analysis based on light spectrum types.

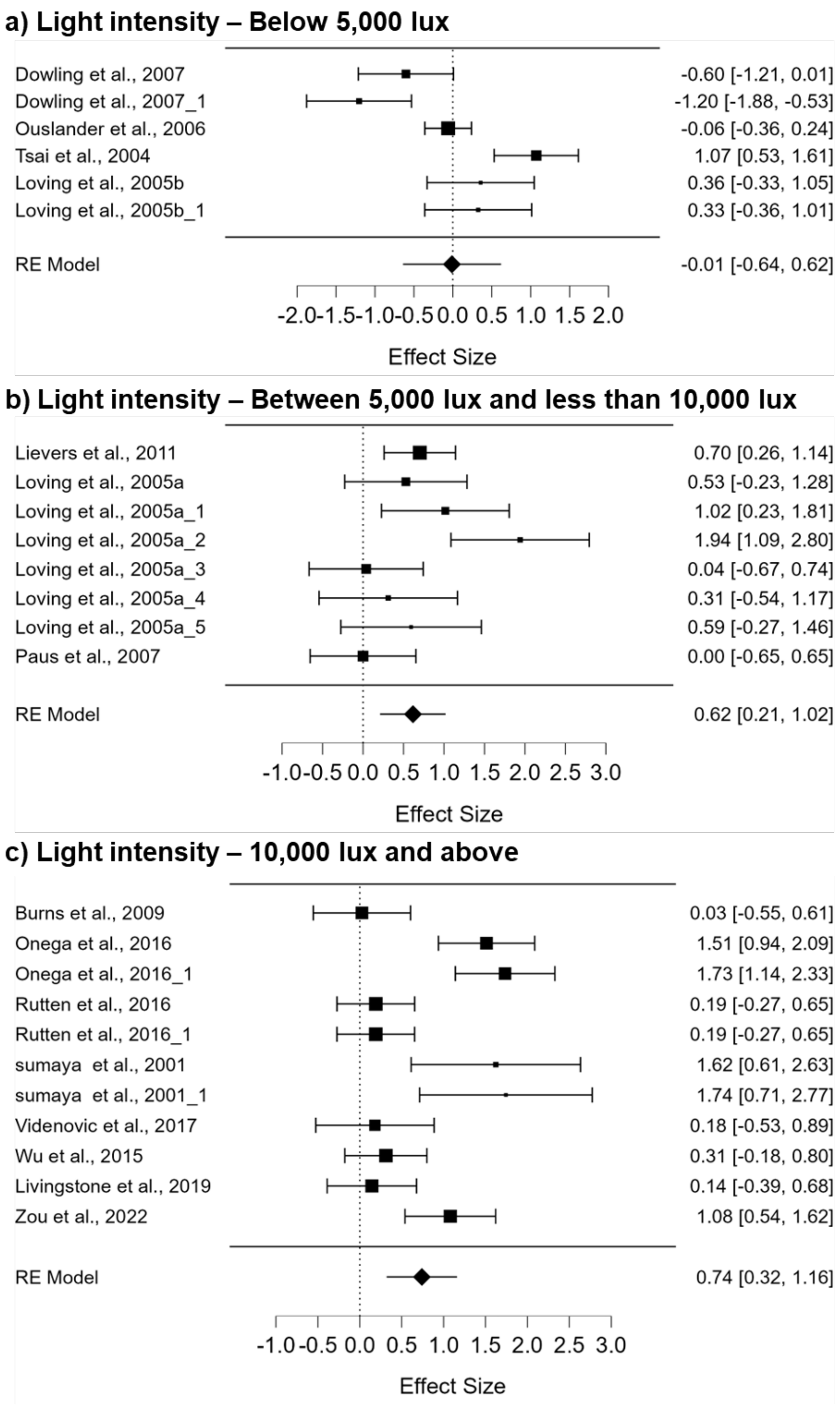

Figure S2. The results of subgroup analysis based on light intensity.

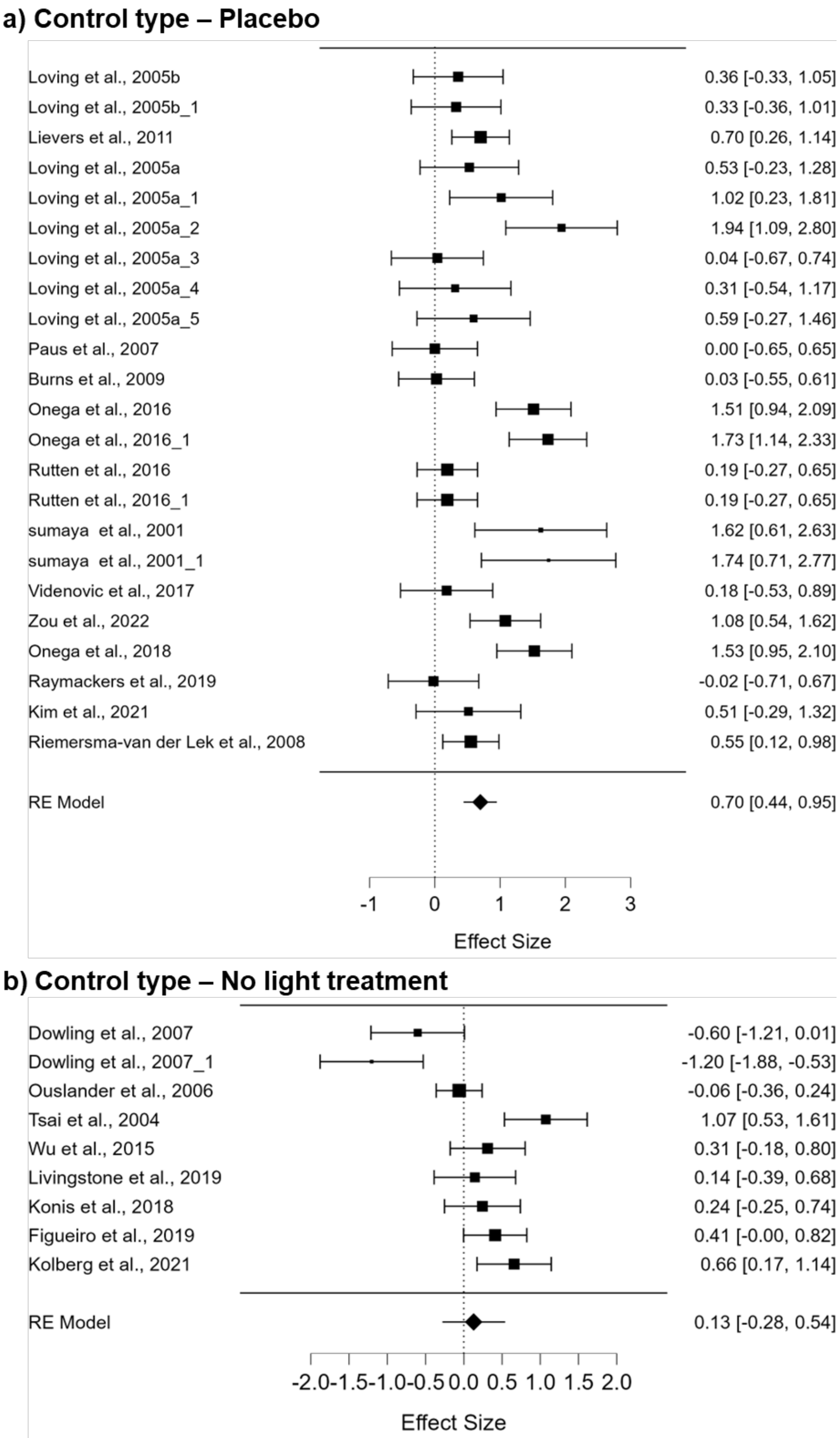

Figure S3. The results of subgroup analysis based on control types
